# Outcomes and complications of open abdomen technique for managing non-trauma patients

**DOI:** 10.4103/0974-2700.62106

**Published:** 2010

**Authors:** Kritaya Kritayakirana, Paul M Maggio, Susan Brundage, Mary-Anne Purtill, Kristan Staudenmayer, David A Spain

**Affiliations:** Department of Surgery, Section of Trauma/Critical Care Surgery, Stanford University School of Medicine, USA

**Keywords:** Damage control, laparotomy, open abdomen, fistula

## Abstract

**Background:**

Damage control surgery and the open abdomen technique have been widely used in trauma. These techniques are now being utilized more often in non-trauma patients but the outcomes are not clear. We hypothesized that the use of the open abdomen technique in non-trauma patients 1) is more often due to peritonitis, 2) has a lower incidence of definitive fascial closure during the index hospitalization, and 3) has a higher fistula rate.

**Methods:**

Retrospective case series of patients treated with the open abdomen technique over a 5-year period at a level-I trauma center. Data was collected from the trauma registry, operating room (OR) case log, and by chart review. The main outcome measures were number of operations, definitive fascial closure, fistula rate, complications, and length of stay.

**Results:**

One hundred and three patients were managed with an open abdomen over the 5-year period and we categorized them into three groups: elective (n = 31), urgent (n = 35), and trauma (n = 37). The majority of the patients were male (69%). Trauma patients were younger (39 vs 53 years; *P < *0.05). The most common indications for the open abdomen technique were intraabdominal hypertension in the elective group (n = 18), severe intraabdominal infection in the urgent group (n = 19), and damage control surgery in the trauma group (n = 28). The number of abdominal operations was similar (3.1–3.7) in the three groups, as was the duration of intensive care unit (ICU) stay (average: 25–31 days). The definitive fascial closure rates during initial hospitalization were as follows: 63% in the elective group, 60% in the urgent group, and 54% in the trauma group. Intestinal fistula formation occurred in 16%, 17%, and 11%, respectively, in the three groups, with overall mortality rates of 35%, 31%, and 11%.

**Conclusion:**

Intra-abdominal infection was a common reason for use of the open abdomen technique in non-trauma patients. However, the definitive fascial closure and fistula rates were similar in the three groups. Despite differences in indications, damage control surgery and the open abdomen technique have been successfully transitioned to elective and urgent non-trauma patients.

## INTRODUCTION

The concept of damage control surgery (DCS) for trauma is now well established in the critically-injured trauma patient.[1] One of the initial descriptions of DCS by Stone *et al*.[2] advocated use of surgical judgment to postpone a definitive surgical procedure in an exsanguinating trauma patient after controlling life-threatening bleeding and closing the abdomen with laparotomy pads packed tightly into the peritoneal cavity. Even when hemorrhage was controlled, these patients often posed other problems secondary to the effects of massive resuscitation. Fietsam *et al.*[3] recognized the complication of abdominal compartment syndrome (ACS) in ruptured aneurysm patients and advocated decompressive laparotomy. Shelly *et al*.[4] described the physiologic effect and hemodynamic parameters following surgical release of increased intra-abdominal pressure in liver transplant patients. Decompressing the abdomen produced an immediate increase in cardiac output that was associated with a decrease in heart rate and an increase in stroke volume. Recognition of ACS as a complication has led to the incorporation of the open abdomen as one of the tenets of DCS, which includes 1) rapid control of bleeding and contamination; 2) temporary abdominal closure; 3) ICU care, with correction of coagulopathy, acidosis, and hypothermia; and 4) delayed definitive surgery.[1,5]

After stabilization in the surgical ICU and ultimate completion of the definitive operation or set of operations, many of these patients remain with an open abdomen as the next major conundrum to be addressed. Many of these patients are intentionally left with a ventral hernia with plans for a delayed repair months later. However, this is being increasingly recognized as a source of major morbidity. Therefore, surgeons have tried out many ways to overcome this problem, including 1) fluid restriction during resuscitation, 2) aggressive postoperative diuresis, 3) use of temporary abdominal closure devices to facilitate sequential abdominal closure, 4) use of biological mesh materials, and 5) component separation techniques to approximate the abdominal wall tissues. All of these methods have been used as adjuncts to facilitate abdominal wall closure during the index hospitalization.[1,5,6]

As modern surgical and critical care capabilities have continued to push the envelope, the concept of abbreviated operation and DCS is now being utilized in the elective and urgent surgical populations with increasing frequency. We have observed use of the open abdomen with increasing frequency in the elective and urgent surgical populations in cases of shock secondary to unanticipated substantial blood loss or fulminant sepsis. In these cases, DCS was implemented with the same principles as in trauma patients: i.e., to stop the vicious cycle of acidosis, hypothermia, and coagulopathy.[5] However, the outcome of open abdomen use in non-trauma patients is less clear. Our purpose was to investigate the outcomes of the open abdomen techniques used in the non-trauma population after damage control laparotomy. We hypothesized that in non-trauma patients use of the open abdomen would 1) more likely be due to a diagnosis of peritonitis, 2) result in a lower definitive fascial closure rate during the index hospitalization, and 3) have a higher fistula rate than in trauma patients.

## METHODS

Study approval was obtained from the Institutional Review Board at our hospital, a tertiary-care, American College of Surgeons (ACS)-verified level-I trauma center. Patients were identified using multiple sources: 1) the Operating Room Information System, 2) medical record review for patients with > 1 laparotomy during a single admission, and 3) the trauma registry. Retrospective data were collected for a 5-year period. All patients receiving laparotomy and who left the OR with an open abdomen or later developed abdominal compartment syndrome requiring decompression were included. Open abdomen was defined as non-approximation of the fascia and skin. Abdominal compartment syndrome was defined as the combination of elevated intra-abdominal pressure with associated new onset of organ dysfunction or failure.[7] Indications for DCS were classified according to Schecter *et al*.[8] Definitive abdominal closure prior to discharge was defined as either 1) primary fascial closure (with or without modified component separation) or 2) placement of a permanent, non-absorbable mesh as a fascial replacement.[1,6]

Patients undergoing laparotomy with resultant open abdomen were categorized into three cohorts: elective, urgent, and trauma. The elective group comprised those who had had an initial operation that was scheduled and intended to be finished in one setting. The urgent group included patients requiring unanticipated and unscheduled emergency operative intervention. The patients in the trauma group were those identified from the institutional Trauma Registry The ‘index operation’ was defined as the operation that led to open abdomen management or abdominal compartment syndrome that needed open abdomen management.

Medical records were reviewed for demographic data, resource utilization, injury severity score (ISS), and APACHE-II score.[9] Details of resource utilization, such as type of procedure and operative details of the index operation, number of operations required secondary to the index operation, hospital length of stay (HLOS), and intensive care unit LOS (ICU LOS), were recorded. Intraoperative data, including that on duration of operation, estimated blood loss, and fluid resuscitation were collected for the index operation, along with details of blood products administered. Outcome data included morbidity (specifically fistula formation), mortality, and status of the abdominal wall at time of discharge.

### Statistical methods

Variables were analyzed using commercially available software (STATA Corporation, College Station, TX). To compare the three cohorts we used the one-way analysis of variance (ANOVA) for continuous variables and the chi square test for categorical variables. *Post hoc* statistical analysis of differences between groups was determined by Student-Newman-Keuls test. Results are presented as mean ± standard error of the mean (SEM). Statistical significance was considered relevant at *P* < 0.05.

## RESULTS

During the 5-year study period, 103 patients were identified as having had open abdomen management at our institute [Table 1]. The majority of the patients were male (69%) in all three cohorts. All patients had a vacuum-type dressing used for management of the open abdomen. The dressing was changed every 48–72 h, either in the OR or in the ICU, depending on the initial indication and patient's status. Indications for initial operative management for elective and urgent surgery patients are shown in Tables 2 and 3. One patient developed secondary abdominal compartment syndrome following lung transplantation complicated by massive bleeding. The total number of celiotomies performed in the trauma cohort during the study period was 253, of which 37 (14%) were managed with an open abdomen. The injury severity score (ISS) for this group was 30 ± 12 (range: 9-57). There is no significant difference in APACHE-II scores in the three groups. The number of abdominal operations per patient (range: 1-12) was not significantly different. Definitive closure rates during the index hospitalization were similar between the three groups [Table 1]. Trauma patients had the lowest mortality rate at 16%.

**Table 1:** Demographic data for open abdomen management (n = 103 patients)

	Elective (n = 31)	Urgent (n = 35)	Trauma (n = 37)	*P* value
Age (mean ± SEM)	53 ± 2.3	53 ± 2.9	39 ± 2.6	<0.001
Female	14 (45%)	11 (31%)	7 (19%)	0.06
APACHE-II (mean ± SEM)	17 ± 1.4	16 ± 1.2	16 ± 1.2	0.82
Mean operations/patient	3.7	3.1	3.1	0.89
Length of ICU stay (mean ± SEM)	27 ± 4	31 ± 3	25 ± 3	0.46
Hospital LOS (mean ± SEM)	48 ± 8.3	39 ± 4.7	34 ± 4	0.26
Definitive closure	19 (63%)	21 (60%)	20 (54%)	0.94
Patients with fistula	5 (16%)	6 (17%)	4 (11%)	0.77
Mortality	11 (35%)	11 (31%)	6 (16%)	0.16

STATISTICAL ANALYSIS WAS WITH THE ONE-WAY ANALYSIS OF VARIANCE (ANOVA) FOR CONTINUOUS VARIABLES AND THE CHI-SQUARE TEST FOR CATEGORICAL VARIABLES

**Table 2:** Indication for surgery in the elective group prior to open abdomen management (n = 31)

Pancreatic tumor	8
Hepatobiliary tumor	5
Colorectal cancer	4
Abdominal aortic aneurysm repair	3
Morbid obesity - gastric bypass	3
Esophageal surgery	2
Renal cell carcinoma	2
Gastric cancer	1
Aortoiliac occlusive disease	1
Peritoneal angiosarcoma	1
Obstetric and gynecologic conditions	1

**Table 3:** Indication for surgery in the urgent group prior to open abdomen management (n = 35)

Peritonitis	10
Orthotopic liver transplantation	5
Bowel obstruction	4
Bowel perforation	3
Necrotizing pancreatitis	3
Bowel ischemia	2
Obstetric and gynecologic conditions	2
Retroperitoneal hemorrhage	2
Ruptured abdominal aortic aneurysm	1
Ruptured hepatocellular carcinoma	1
Toxic megacolon	1
Secondary abdominal compartment syndrome	1

The indications for use of the open abdomen, which may be multiple, are shown in Table 4. Intra-abdominal hypertension was the most common indication in the elective group. Most commonly, this was a complex upper abdominal operation that was complicated by excessive blood loss and significant resuscitation. The indication of severe abdominal infection was significantly higher in the urgent cohort (*P = *0.005). The trauma group, as expected, required damage control laparotomy most commonly (*P = *0.02).

**Table 4:** Indications for open abdomen management (may be 1/patient)

	Elective	Urgent	Trauma	*P* value
Severe abdominal infection	7	19	3	0.005
Mesenteric ischemia	2	3	1	0.6
Necrotizing abdominal wall infection	7	1	0	0.004
Damage control laparotomy	12	8	28	0.02
Intra-abdominal hypertension	18	10	8	0.09

CHI-SQUARE TEST WAS USED FOR CATEGORICAL VARIABLES

During the index operation leading to open abdomen, elective surgery patients were more likely to have longer operative times, less fresh frozen plasma and platelet utilization, and more colloid administration [Table 5]. Patients undergoing urgent operation had significantly lower operative time than those in the elective and trauma cohorts (*P = *0.001). Estimated blood loss was similar among cohorts and the amount of crystalloid and packed red cell used in the OR was not significantly different between the three cohorts [Table 5].

**Table 5:** Intraoperative record for the index operation leading to open abdomen

	Elective (n = 31) (Mean ± SEM)	Urgent (n = 35) (Mean ± SEM)	Trauma (n = 37) (Mean ± SEM)	*P* value
OR time (min)	168 ± 16*	106 ± 10	137 ± 8	0.001
Estimated blood loss (ml)	2018 ± 287	1690 ± 317	1875 ± 104	0.656
Crystalloid (ml)	4645 ± 509	4181 ± 387	4000 ± 215	0.471
Colloids (ml)	1041 ± 100	958 ± 96	525 ± 30*	<0.001
Packed red cell (units)	9.14 ± 1	7 ± 1	7.5 ± 0.5	0.213
Fresh frozen plasma (units	4.8 ± 0.5*	7.6 ± 1	7.3 ± 0.5	0.013
Platelets (packs)	1.5 ± 0.1	1.75 ± 0.2	3 ± 0.2*	<0.001

ONE WAY ANALYSIS OF VARIANCE (ANOVA) WAS USED FOR STATISTICAL ANALYSIS

## DISCUSSION

When we initially began this analysis, our intent was to compare open abdomen outcomes in non-trauma and trauma patients. However, it quickly became apparent that the non-trauma patients comprised two distinct patient populations. The first was the elective case gone awry — most typically, complex upper gastrointestinal tract surgery complicated by unexpected significant blood loss. The second cohort was the urgent case where, most commonly, the surgeon was aware preoperatively of significant intra-abdominal pathology. Confirming a portion of our hypothesis, we did find intra-abdominal infection as a common indication for use of the open abdomen technique in the urgent group, while intra-abdominal hypertension was the most common indication following elective surgery.

Our data suggest that, despite these differences in etiology, use of the open abdomen technique following DCS in elective or urgent operations in non-trauma patients had similar results to that in trauma patients as measured by outcomes (fistula rate: 11-17% and fascial closure: 54-63%) and resource utilization (number of operations and ICU LOS); this was despite the fact that the trauma patients were significantly younger. Overall, we were pleased by the relatively low fistula rate but disappointed with the fascial closure rate. There are several possible explanations for this. First, these patients were managed by different types of surgeons, including trauma/acute care surgeons, but also surgical oncologists and colorectal and vascular surgeons. As such, there was no formalized approach for management of these patients. Finally, this study spans a transition stage when the use of planned ventral hernia[6] was being phased out and replaced by attempts at serial abdominal closure. Based on the data from the work of others,[10–12] we have adopted a more aggressive approach to early fascial closure. The open abdomen is initially managed with a commercially prepared sponge device (VAC, KCI International, San Antonio, Texas). Once the patient has completed resuscitation and is thermodynamically stable, we begin aggressive diuresis to facilitate closure. The dressing is changed every 48 h, with sequential attempts at fascial closure with each dressing change. If after several changes we are not making satisfactory progress with the vacuum dressing and serial closure, a Wittmann patch (Star Surgical Inc., Burlington, WI) is used.[10]

Converting from an elective surgical mindset to one of damage control can be very challenging. No surgeon starts an elective operation planning to leave the OR with the abdomen packed and left open. This is a humbling experience for the surgeon and is cause for some reluctance to use this technique. Yet, in this series, the average operative time for elective cases was just under 3 h, which did not seem extraordinarily long, given the complex nature of these operations. This suggests that prolonged attempts at definitive surgery were abandoned and the concepts of abbreviated operation and DCS were adopted relatively quickly once significant blood loss (on average 2l) was encountered. The shorter operative time in the urgent cohort may be due to the attending surgeon's perspective on the outcome for the patient preoperatively. In fact, as we transition to an acute care surgery model, many of these procedures were performed by the ‘trauma surgeons,’ who likely entered into the procedure with a plan to terminate the operation once bleeding and contamination were controlled.[13–15]

Although the estimated blood loss, packed red blood cells transfused, and crystalloid fluid given were similar in all groups, fresh frozen plasma was used more in the urgent and trauma groups and trauma patients received more platelets. This suggests that, although some of the concepts of DCS have been adopted, the current practice of damage control resuscitation has not made the transition. Many trauma transfusion protocols now advocate a 1:1:1 ratio of packed red blood cells, fresh frozen plasma, and platelets.[16,17] This terminology refers to the older method of dispensing a ‘six-pack’ of pooled platelets. Most centers now dispense a ‘unit’ of apheresis platelets from a single donor which contains roughly an equivalent amount of platelets as a “six pack”. In this study we used the older terminology to approximate the 1:1:1 ratio. In this respect, the resuscitation in the elective surgery patient was deficient in both fresh frozen plasma and platelets, while both the urgent and trauma patients should have received more platelets.

The need to perform DCS and use the open abdomen technique in elective surgery patients was associated with a significant mortality rate (35%). This likely reflects the significant blood loss and underlying comorbidities (24/31 had cancer). However, it is possible that failure to achieve fascial closure and the development of an enterocutaneous fistula further compromised the nutritional status of these patients and contributed to the high mortality rate. Fistula formation in these patients is often the so-called entero-atmospheric fistula, which is very difficult to control. These patients face months of pain, complex wound care, and compromised nutritional status, which is then followed by major abdominal wall reconstructive surgery. We have been concerned with the morbidity associated with these secondary procedures. Thus, we have adopted a much more aggressive approach to obtaining fascial closure during the index hospitalization. A variety of techniques have been described, each with inherent benefits and weaknesses.[10–12] Each may have a place in the management of the open abdomen at different stages. Thus, the surgeon must remain open-minded and flexible. Ideally, there must be a concerted commitment to obtain fascial closure during the first 7–10 days.

DCS and the open abdomen techniques are valuable tools for the management of patients with acidosis, hypothermia, and coagulopathy. This is a very resource-intensive decision. These patients require several operations, the fistula rate is 5-15%,[10–12,15] and the patients have prolonged hospital stays. Thus, these complex patients required concerted and consistent care by a dedicated team (surgeons, intensivists, and anesthesia and OR staff, etc).[10,15] However, surgeons should not be reluctant to use this technique selectively in urgent situations or elective operations with complications, as this approach may be reduce mortality in these critically ill patients.[15]
